# Osteogenesis and angiogenesis of a bulk metallic glass for biomedical implants

**DOI:** 10.1016/j.bioactmat.2021.06.018

**Published:** 2021-06-20

**Authors:** K. Sun, R. Fu, X.W. Liu, L.M. Xu, G. Wang, S.Y. Chen, Q.J. Zhai, S. Pauly

**Affiliations:** a Institute of Materials, Shanghai University, Shanghai, 200444, China; b Department of Neurology, Tongren Hospital, School of Medicine, Shanghai Jiao Tong University, Shanghai, 200336, China; c Sports Medicine Department of Huashan Hospital, Fudan University, Shanghai, 200040, China; d University of Applied Sciences Aschaffenburg, Würzburger Straße 45, D-63743, Aschaffenburg, Germany

**Keywords:** Metallic glasses, Bio-corrosion, Biocompatibility, Osteogenesis, Angiogenesis

## Abstract

Implantation is an essential issue in orthopedic surgery. Bulk metallic glasses (BMGs), as a kind of novel materials, attract lots of attentions in biological field owing to their comprehensive excellent properties. Here, we show that a Zr_61_Ti_2_Cu_25_Al_12_ (at. %) BMG (Zr-based BMG) displays the best cytocompatibility, pronounced positive effects on cellular migration, and tube formation from *in-vitro* tests as compared to those of commercial-pure titanium and poly-ether-ether-ketone. The *in-vivo* micro-CT and histological evaluation demonstrate the Zr-based BMG can significantly promote a bone formation. Immunofluorescence tests and digital reconstructed radiographs manifest a stimulated effect on early blood vessel formation from the Zr-based BMG. Accordingly, the intimate connection and coupling effect between angiogenesis and osteogenesis must be effective during bone regeneration after implanting Zr-based BMG. Dynamic gait analysis in rats after implanting Zr-based BMG demonstrates a tendency to decrease the pain level during recovery, simultaneously, without abnormal ionic accumulation and inflammatory reactions. Considering suitable mechanical properties, we provide a realistic candidate of the Zr_61_Ti_2_Cu_25_Al_12_ BMG for biomedical applications.

## Highlights

• Zr-based metallic glass can promote a bone formation and achieve a stimulated effect on early blood vessel formation.
• The pain level during recovery in rats is decreased after implanting Zr-based metallic glass from dynamic gait analysis.
• A realistic candidate of the Zr-based metallic glass for biomedical applications is provided.

## Graphical abstract

**Figure:**
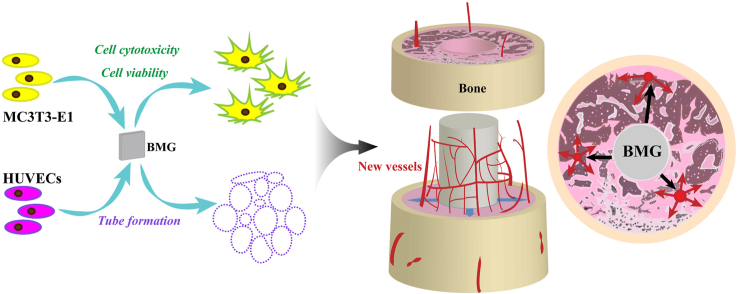

## 1 Introduction

Due to remarkably mechanical performances and superior corrosion resistance, bulk metallic glasses (BMGs), as a kind of glassy materials, are arousing wide concerns from numerous fields in the past decade [[1], [2], [3]]. In the area of biomedical application, especially in biological implants, such as bone-fracture fixation devices, artificial joint prosthesis, or dental implants, biocompatibility and mechanical behavior are two crucial factors to determine the suitability of a given material. Biocompatibility is fundamental for avoiding inflammations, complications, and other abnormal reactions of the organism to the implanted material. On the premise of biocompatibility, the mechanical properties are equally important [4]. The fracture of grafts due to insufficient strength, the stress shielding phenomenon caused by excessively high elastic modulus, and debris related to a poor wear resistance could lead to various immune responses or unexpected osteoatrophy [5]. Therefore, it is vital to develop appropriate materials with long-term safety and stability, which can prevent the need for revision surgeries and also effectively reduce unnecessary suffering of patients during recovery [6].

Metals play an indispensable role in orthopedic applications. Commercial materials, such as Co-Cr alloys, 316L stainless steel, and titanium, are commonly and widely used biodevices in clinic. They are accepted by the body environment but are not ideal for biological applications since some properties needed to be improved, e.g., toxic effect [7], and poor tribology [8]. Ti-based alloys are widely applied in regenerative medicine, which can improve cell cytocompatibility [11,12]. Accordingly, Ti-based BMGs have been investigated [9,10] owing to their enhanced wear resistance [13] compared to Ti-based alloys, as well as the more excellent mechanical and chemical characteristics inherently originated from the glassy structure. However, Ti-based BMGs' Young's modulus of around 80–120 GPa are substantially higher than that of human bone of 10–40 GPa [4,14]. In contrast, Zr-based BMGs attract more attention due to their low Young's modulus (50–89 GPa) [3,4], which make them a potentially desirable material serving for osteosynthesis [15,16].

A Zr_61_Ti_2_Cu_25_Al_12_ BMG (ZT1) becomes more innovative since its superior biocompatibility with modern imaging techniques, such as a magnetic-resonance imaging (MRI) technique [17], and a biologically mechanical property [18]. An *in-vivo* oral mucosa irritation measurement indicates that the ZT1 has a possibility to be used as a dental implant [19]. It also achieved a considerable response regarding its osteogenic activity [20], which is in agreement with other BMGs, such as Zr_70_Ni_16_Cu_6_Al_8_ [21], and Zr_60.14_Cu_22.31_Fe_4.85_Al_9.7_Ag_3_ BMGs [3]. Particularly, Li et al. found that the ZT1 could stimulate an expression of genes related to induce angiogenesis and permeabilization of blood vessels [19]. However, there is no direct evidence from experiments that the ZT1 can simultaneously enhance angiogenesis with promoting osteogenesis, especially *in-vivo*, which seriously prohibits the application of the ZT1.

**Fig. 1:**
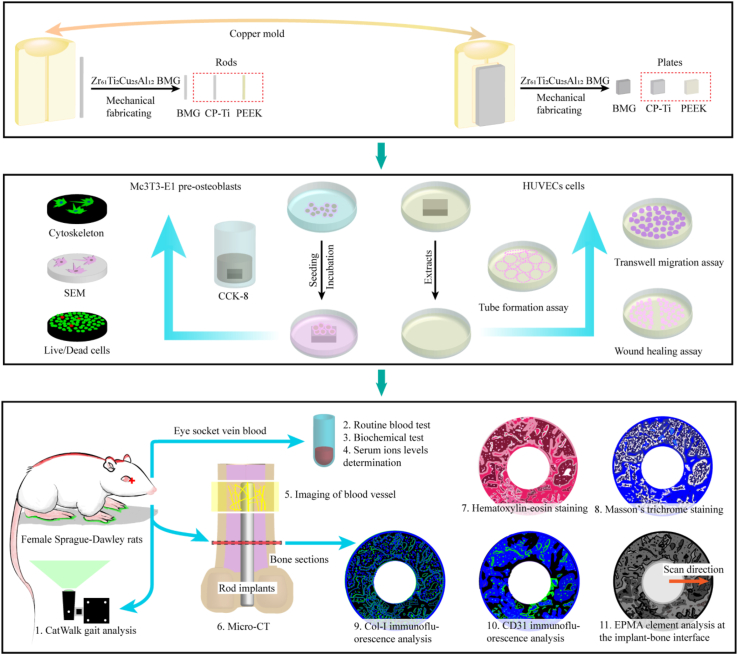
The flowchart of material preparation, bio-corrosion test, *in-vitro* measurements, and *in-vivo* evaluations.

In the present work, the ZT1 is subjected to comprehensive *in-vitro* and *in-vivo* evaluations to explore its physicochemical and biological properties, as the flowchart shown in Fig. 1 and S1. A pure titanium (CP-Ti) and a poly-ether-ether-ketone (PEEK) are chosen for comparison since they are golden standards for medical assessment of implanted metals and polymers, respectively. The aim of this study is to evaluate the chemical stability and biological safety of the ZT1 for clinical requirements. Moreover, the profile of systemic angiogenesis analysis of the ZT1 is mapped, which plays a critical role in skeletal development and repair. The reasons for the observed better osseointegration of the ZT1 at different stages of tissue recovery are discussed. Our work provides realistic evidences for the biocompatibility of the ZT1, and thus reveals a prospective candidate for orthopedic applications.

## 2 Materials and methods

### 2.1 Materials preparation and characterization

The master alloy of Zr_61_Ti_2_Cu_25_Al_12_ was prepared by arc-melting a mixture of pure elements (purity ≥ 99.99%) in a titanium-gettered atmosphere, which was followed by suction casting in a copper mold to form rod-shaped samples with a diameter of 2 mm, and 2-mm-thick plate samples. The rods were mechanically polished to 1.1 mm in diameter and 9.8 mm in length, and then were subsequently applied in an immersion test and *in-vivo* evaluations. The plates were prepared into square sheets with an 8-mm side, and used for a hydrophilic characterization, a bio-corrosion test, and *in-vitro* cell measurements. The materials for comparison, CP-Ti (Beijing Goodwill Metal Co. Ltd., Beijing, China) and PEEK (Nanjing Welling Shuangxing New Material Technology Co. Ltd., Nanjing, China), were prepared into the same geometric size to that of the ZT1. Samples were polished to a mirror surface and cleaned successively by acetone and alcohol. Sterilization by autoclaving was necessary before the *in-vitro* and *in-vivo* evaluations.

The glassy structure of the ZT1 was examined by X-ray diffraction (XRD) in a D\max-2550X diffractometer with a Co-K_α1_ radiation (*λ* = 1.5406 Å) generated at 40 kV. The surface roughness was measured in an APHENOMTMG2 (FEI company) scanning-electron microscope (SEM) cooperated with a three-dimensional profilometer (Phenom-World). The hydrophilic characterization of the ZT1, CP-Ti, and PEEK was achieved by a video contact angle measurement system (JC2000D3, Zhongchen Technology, Shanghai, China). A 4-μL droplet of deionized water as a testing medium was used to measure the surface contact angle of each material. All tests were repeated at least six times to ensure reproducibility.

### 2.2 Corrosion tests

According to the ASTM-G31-72 standard, the rod-shaped samples (the ZT1 and the CP-Ti) were immersed in a 15 mL simulated body fluid (SBF) solution at a constant temperature of 37 °C for 1, 2, 4, and 6 weeks. The SBF is a solution buffered with ion concentrations closing to those of human plasma. An immersion in the SBF can evaluate the performances and properties of various materials *in-vitro*. After the immersion, inductively coupled plasma mass spectrometry (ICP-MS; ThermoScientific, USA) was employed to measure the concentrations of ions released from the ZT1 and the CP-Ti.

The bio-corrosion behaviors of the ZT1 and the CP-Ti were analyzed in an electrochemical workstation (CHI600C, Shanghai Chenhua Instrument Co. Ltd., China) in the SBF at room temperature. Electrochemical polarization measurements were conducted in a three-electrode cell using a platinum counter electrode and a saturated calomel reference electrode (SCE). The sample was initially placed in the SBF for 3600 s in order to adapt to the environment, and then polarization test was carried out at a scanning rate of 0.01 V/s. After the electrochemical test, samples were cleaned, and X-ray photoelectron spectroscopy (XPS; ESCALAB250Xi) was conducted to identify the chemical composition on the surface of the ZT1 and the CP-Ti. The etching experiment was lasted for 1900 s in an AXIS-Ultra instrument from Kratos Analytical with an ion energy of 1000 eV, etching rate of 0.09 nm/s, and raster size of 3 mm. Binding energies were calibrated using C 1s hydrocarbon peak at 284.80 eV to compensate the surface charge effect. Further analysis was processed in Avantage software (Thermo Scientific).

### 2.3 In-vitro measurements

#### 2.3.1 Cytocompatibility measurements with MC3T3-E1 pre-osteoblast

Murine MC3T3-E1 pre-osteoblasts (The Cell Bank of Type Culture Collection of Chinese Academy of Sciences, Shanghai, China) were used for the *in-vitro* assessment of cytocompatibity. It is a kind of pre-osteoblasts, which is sensitive to the surroundings, and without such strong survivability. The cells were cultured in an alpha minimum essential medium (α-MEM, Gibco, Carlsbad, CA, USA) supplemented with 10% fetal bovine serum (FBS, Gibco) and 1% penicillin-streptomycin (Gibco) under standard conditions, i.e., 37 °C, 95% humidity, and 5% CO_2_. The culture medium was refreshed every two days. Third-passage of MC3T3-E1 pre-osteoblasts were seeded on samples for the assessments of viability, morphology, adhesion, and proliferation experiments.

Cell viability of MC3T3-E1 pre-osteoblasts was analyzed by a LIVE/DEAD Cell kit (KeyGEN Biotech, Beijing, China). After culturing for 24 h, all samples were transferred to a new 24-well culture plate, washed twice with phosphate buffered saline (PBS), and then stained with the 1 mL combination dye of propidium iodide and the Calcein-AM in the PBS at room temperature for 30 min. Afterwards, the samples were washed twice with the PBS. The distributions of living cells and dead cells on the surface of the investigated samples were examined by a confocal laser scanning microscope (CLSM, C2+, Nikon, Japan). The percentages of living and dead cells were calculated by a software of ImagePro Plus 6.0. For each kind of material, five samples of each material were examined to ensure the accuracy of the experiments.

The samples were immersed in total medium for 10 min in the incubator, and then MC3T3-E1 pre-osteoblasts were seeded on the surface of samples with a density of 1 × 10^5^ cells/mL in 24-well culture plates. After culturing for 6 h and 24 h, the samples were washed twice with the PBS, and then fixed with 2.5% glutaraldehyde (Solarbio Company, Beijing, China) for 2 h at 4 °C. After the dehydration in an ascending series of ethanol, the samples with cells were subjected to drying, and then coated by golden. The morphology of MC3T3-E1 pre-osteoblasts that were seeded on the investigated materials was examined by the SEM (HITACHI SU-1500). The cells were marked by purple. Cell area (CA) was calculated in the ImagePro Plus 6.0 software. The cell morphology test was repeated three times.

After co-culturing for 24 h, the samples were washed twice by the PBS, and then fixed with 4% paraformaldehyde solution for 15 min and permeabilized with 0.1% Triton X-100 for 10 min. Afterwards, the cell filamentous actins were labeled with 1 μg/mL phalloidin-FITC (Solarbio) for 30 min, while the cell nuclei were treated with 2 μg/mL 4′, 6-diamidino-2-phenylindole (DAPI, Solarbio) for 5 min. The fluorescently stained cells were observed by a confocal laser scanning microscope (CLSM, C2+, Nikon, Japan). Projected cell area (PCA) and integrated optical density (IOD) were calculated in the ImagePro Plus 6.0 software. For each kind of material, six samples were repeated to exclude the occasional cases.

The cell cytotoxicity was examined by a Cell Counting Kit-8 assay (CCK-8, Dojindo, Kumamoto, Japan), which was carried out in 96-well plates. 1 mL of fresh α-MEM containing 10% CCK-8 solution was added to each well. After 3 h, 100 μL of the mixed medium was transferred to a 96-well plate. The value of optical density (OD) was measured at a wavelength of 450 nm with a microplate reader (Infinite F50, TECAN, Switzerland). The measurements were repeated three times.

#### 2.3.2 In-vitro cell (HUVECs, human umbilical endothelial cells) angiogenesis measurements

Human umbilical endothelial cells (HUVECs, American Type Culture Collection, Manassas, VA, USA) were used to analyze the *in-vitro* cell angiogenesis. They were cultured in a high-glucose Dulbecco's Modified Eagle's Medium (DMEM, Gibco) supplemented with the 10% FBS (Gibco) and 1% penicillin/streptomycin (Gibco) under conditions of 37 °C, 95% humidity, 5% CO_2_. The culture medium was refreshed in every 2 days. Third-passage cells were used for an inoculation in the subsequent experiments. For each measurement, 6 samples were taken to ensure the experimental accuracy.

The ZT1, CP-Ti, and PEEK were immersed into the DMEM supplemented with the 10% FBS for 72 h under the conditions of 37 °C, 95% humidity, 5% CO_2_ with a fixed concentration of 0.2 g/mL for preparing extracts according to ISO 10993 Part 12. Three kinds of extracts including ZT1, CP-Ti, and PEEK for the following wound healing assay, transwell migration assay, and tube formation assay were collected without any filtration. Each assay was repeated six times for each sample to ensure the experimental accuracy.

HUVECs were seeded at a density of 1 × 10^5^ cells/well in a 24-well culture plate and cultured until they reached a confluence of about 100%. Subsequently, linear wounds were made with a sterile 1-mL plastic pipette tip. Next, floating cells and debris were removed with PBS, and the diluted extract corresponding to materials was added with the concentration of 12.5%. After an additional 24 h, the migration of HUVECs into the wound was assessed by an optical microscopy. The denuded area was calculated with the ImagePro Plus 6.0 software and expressed as the percentage of wound closure.

The transwell migration assay was conducted by utilizing transwell inserts with an 8-μm pore size filter (BD Biosciences, USA). HUVECs with a density of 2 × 10^4^ cells/well were seeded in the culture medium into the upper chamber of the insert precoated with Matrigel, and 700 μL of diluted extract mentioned above was added to the lower chamber. After 24 h of incubation, HUVECs were fixed with 75% ethanol and stained with crystal violet (Solarbio). Then, HUVECs on the top membrane surface were wiped off, and HUVECs on the lower surface were fixed in 4% paraformaldehyde, stained with 0.1% crystal violet, and photographed in a microscope. For quantitative analysis, the number of cells invading through the Matrigel coated chamber was counted in the ImagePro Plus 6.0 software.

HUVECs were pretreated with the diluted extract for 24 h. The cells were harvested and seeded at a density of 4 × 10^4^ cells/well on growth factor-depleted Matrigel (BD Biosciences, USA) in 48-well plates and cultured for 24 h. The original culture medium was subsequently replaced by the diluted extract mentioned above. After 4 h’ co-culturing under standard conditions (37 °C, 95% humidity, 5% CO_2_), the tube structures formed in the gel were photographed in a microscope. The tube formation parameters were measured by WIMASIS software (WimTube, WIMASIS, Córdoba, Spain).

### 2.4 In-vivo measurements

All experimental protocols involving animals were approved by the Institutional Animal Care and Use Committee of Fudan University complied with the “Guide for the Care and Use of Laboratory Animals”. Rats femoral condylar insertion model was utilized for *in-vivo* evaluations. A total of 90 female Sprague-Dawley rats were used (SLAC, Shanghai, China), which were randomly divided into the Cp-Ti, PEEK and ZT1 groups. (8-weeks old, 250 g, 30 rats in each group).

After general anesthesia by an intraperitoneal injection of chloral hydrate, the right leg of each rat was shaved and cleaned with povidone iodine. Figure S1 shows the *in-vivo* surgery process. As shown, a medial parapatellar arthrotomy was performed to expose the right intercondylar fossa, which is the deep notch between the medial and lateral femoral condyles. A 10-mm long bone tunnel was drilled through the intercondylar fossa with a Ф 1.2 mm Kirschner wire. Subsequently, the sample was inserted into the tunnel. After rinsing the joint with PBS, the fascia and skin were closed in layers. The rats were euthanized two, four, and six weeks after surgery by the administration of an overdose of chloral hydrate.

#### 2.4.1 High-resolution micro-CT evaluation

After euthanasia, the femurs with implants were isolated, fixed in 4% paraformaldehyde, and scanned using a high-resolution micro-CT (Skyscan 1176, Bruker, Belgium). The scanning parameters were set as: Cu 0.1 mm filter, 2 × 2 camera binning, 18 μm spatial resolution, 90 kV, 270 μA, 360° rotation, and 1 average frame at every 0.4° angle step. The reconstruction of images was accomplished using the NRecon software version 1.6 (Bruker, Belgium), with ring artefact reduction of 1, 25% of beam hardening correction, smoothing of 3, and the output attenuation values within the range of 0.006–0.1. Reconstructed data were further analyzed by CT-Analyzer through controlling the minimum grey threshold value of 30 and the maximum of 255. The region of interest (ROI) was defined as a column at the center of the bone tunnel of 1.6 mm in diameter, and it was 1.5 mm above the growth plate of the condyles. 50 axial images were reconstructed into a 3D image which was used to measure object volume (BV mm^3^), percent object volume (BV/TV %), total ROI surface (TS mm^2^), object surface (BS mm^2^), intersection surface (i.S mm^2^), object surface/volume ratio (BS/BV 1/mm), object surface density (BS/TV 1/mm) and trabecular pattern factor (Tb.Pf 1/mm). For each material, five samples were assessed to ensure the experimental accuracy.

#### 2.4.2 Histological analysis

After fixation in 4% paraformaldehyde for 48 h, the femurs were subsequently decalcified in 10% EDTA (PH ≈ 7.2) for 4 weeks. Afterwards, the inserted implants were carefully removed, and the femurs were dehydrated and embedded in paraffin. Tissue sections were cut to 8-μm-thick slides, which were mounted on glass slides, and subjected to hematoxylin/eosin (HE) and Masson's trichrome staining. All the slides were scanned using a digital slide scanner (NanoZoomer S60, Hamamatsu, Japan). The photographs were obtained by means of NDP Scan 3.2.6 software. The bone area ratio (B.Ar/T.Ar), which was calculated the percentage of bone tissue to the whole section, was determined using ImagePro Plus 6.0 software and compared among various materials. The measurements were repeated with four specimens.

#### 2.4.3 Immunofluorescence analysis and imaging of blood vessels

Immunostaining was performed using a standard protocol [22]. Specifically, bone sections were incubated with primary antibodies at 4 °C overnight. The used primary antibodies were Mouse monoclonal anti-human Col-I (1:200, ab6308, Abcam, USA) and mouse monoclonal anti-human CD31 (1:200, ab9498, Abcam, USA). Appropriate Alexa Fluor-coupled secondary antibodies (1:400, Molecular Probes, Life Tech, USA) were used to detect the primary antibodies. After the final wash, the nuclei were counterstained with DAPI (1:1000, D1306, Life Tech, USA) in PBS before imaging. Eventually, coverslips were sealed with nail polish, and then the sections were analyzed under CLSM (Nikon). Signal intensities were quantified using ImagePro Plus 6.0 software. Five samples of each material were assessed to increase reliability of analyses.

Two weeks after surgery, three rats from each group were euthanized by an intraperitoneal injection of chloral hydrate. Their thoracic and abdominal cavity were opened, and the inferior vena cava was severed. A needle was inserted into the left ventricle, and the vasculature was flushed with 0.9% normal saline containing heparin sodium (100 U/mL). Then the inferior vena cava was clamped, and the vasculature was injected with 50 mL of silicone rubber compound (Microfil MV-122; Carver, MA, USA). Adequate filling of the arteries was assessed when the vessels of the gut, liver, and the lower limbs appeared filled with the yellow silicone rubber. The silicone rubber was left to polymerize at room temperature for 30 min, and the samples were stored overnight with the ambient temperature of 4 °C. Afterwards, the femurs were dissected and fixed in 4% paraformaldehyde for another 48 h. The fixed femurs were decalcified in 10% EDTA (Sigma Aldrich) for 4 weeks. Images were collected with a high-resolution micro-CT imaging system (Skyscan 1176). Micro-CT with a spatial resolution of 9 μm was used to evaluate the blood vessels formation in femoral samples at 45 kV, 556 μA and Al 0.2 mm filter. A NRecon software and CT-Analyzer were used to reconstruct the 3D angiographic architecture, the minimum grey threshold value was set at 170 and the maximum was 255. All tests were repeated with three specimens.

#### 2.4.4 In-vivo inductively coupled plasma and hematological analysis

Six weeks after surgery, femurs from each group were dehydrated in an ascending series of ethanol and embedded in polyester resin. Each femur was sectioned perpendicular to its longitudinal axis using a diamond saw (Secotom-60, Struers, Denmark) with a low feed speed of 0.020 mm/s. The corresponding thin sections with 2 mm in thickness across the bone-implant interface were obtained. The obtained sections were managed step by step. They were first rinsed under running water, ultrasonically cleaned for 3 min with ethanol and deionized water, and then dried in air. Next, the sections were coated with a thin layer of silver for subsequent observation. X-ray intensities for Zr, Ti, Cu, Al, and Ca were analyzed across the implant-bone interface by means of a scanning probe microscope connected to the electron probe X-ray microanalysis (SEM-EPMA, SPM-9700, Shimadzu, Japan).

Blood analysis was performed 2, 4, and 6 weeks after the surgery. The rats were deprived of food and water for 24 h, and then, 0.5 mL of blood was collected from eye socket vein for routine blood test through a HV950FS hemocytometer (Erba Diagnostics, Germany). Two milliliters of blood were collected for biochemical test using a Clinical Analyzer (HITACHI, Tokyo, Japan) and 2.5-mL of blood was collected for determining the serum ionic concentration of Zr^2+^, Ti^2+^, Cu^2+^, and Al^3+^ via inductively coupled plasma-mass spectroscopy (ICP-MS, ICAP-Q, Thermo Fisher, USA).

#### 2.4.5 Gait analysis

Gait analysis was performed by means of the CatWalk automated gait analysis system (Noldus Technology Company; software version XT 10.5.505). The analysis was carried out at 0 (before implantation), 1, 2, and 3 weeks after implantation. A camera was set at 60 cm beneath the cleaned glass walkway. The background image was captured before recording each trial. The video recording of paw prints was automatically triggered once the rat entered the ROI voluntarily. Additionally, the walkway was cleaned if a rat left urine or feces behind. Recorded runs were accepted as compliant behaviors with a moderate walking speed variation less than 60%. Paw print was auto-classified as left front (LF), right front (RF), left hind (LH), and right hind (RH) through the built-in software. Gait parameters were presented as ratios between the target (operated) side and the contralateral side in order to minimize the confounding influences from body weight and paw size. Five rats per group were analyzed at each point in time.

### 2.5 Statistical analysis

All data are depicted as the form of mean values with standard deviations (SD). A Tukey post-hoc test was adopted to conduct comparisons between pairs of group after one-way Analysis of Variance (ANOVA) for a single time point and two-way ANOVA for multiple time points. A *P* value < 0.05 was considered statistically significant, where 0.01 ≤ *P* < 0.05 marked by *, 0.001 ≤ *P* < 0.01 marked by **, 0.0001 ≤ *P* < 0.001 marked by *** and *P* < 0.0001 marked by ****, shown in the histogram graphic. Statistical analysis was conducted in GraphPad Prism Software (Version 6.0c, GraphPad Software Inc., San Diego, CA, USA).

## 3 Results

### 3.1 Microstructural and hydrophilic characterization

**Fig. 2:**
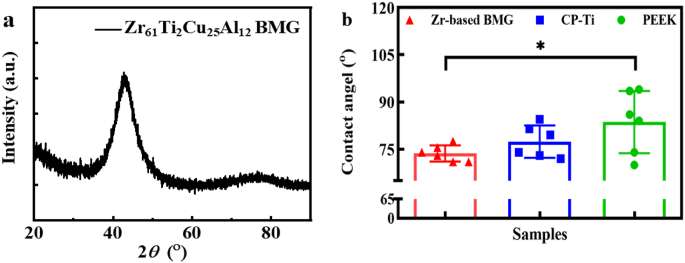
Microstructural and hydrophilic characterization of the investigated materials. a The XRD pattern of the Zr_61_Ti_2_Cu_25_Al_12_ BMG. **b** The wettability, i.e. contact angle of the investigated materials. Significant differences in contact angle take place between the Zr-based BMG and PEEK since *P = 0.049. A *P* value < 0.05 was considered statistically significant, where 0.01 ≤ *P < 0.05, 0.001 ≤ **P < 0.01, 0.0001 ≤ ***P < 0.001, and ****P < 0.0001 shown in the histogram graphic.

The XRD pattern plotted in Fig. 2a exhibits a typical broad scattering signal, revealing the amorphous structure of the ZT1. The surface roughness of three materials observed by SEM displays no significant differences (Figs. S2a – b). The hydrophilicity of various materials is estimated based on contact-angle measurements (Fig. S2c), giving the average values of contact angles of 73.7 ± 2.6°, 77.4 ± 5.1° and 83.6 ± 9.9° for the ZT1, CP-Ti and PEEK, respectively (Fig. 2b). By means of comparison, the ZT1 displays a more hydrophilic surface characteristic.

### 3.2 Immersion test and corrosion behavior

The results of ICP-MS, i.e., the cumulative ionic concentrations released by the ZT1 and CP-Ti in the SBF solution as a function of immersing time are shown in Fig. S3a. No significant differences in the cumulative ion concentration at different times suggests that metal ions are generally released to the SBF solution within the first week. Meanwhile, the ion concentration of Cu is obviously higher than that of other elements. The electrochemical measurements (Fig. S3b) show that the ZT1 has better corrosion resistance than the CP-Ti in physiological solutions due to the formation of protective oxide films proved by XPS analysis (Figs. S3c – l). It is obvious that the oxide layer can effectively block the metal ions during immersing.

### 3.3 In-vitro measurements

**Fig. 3:**
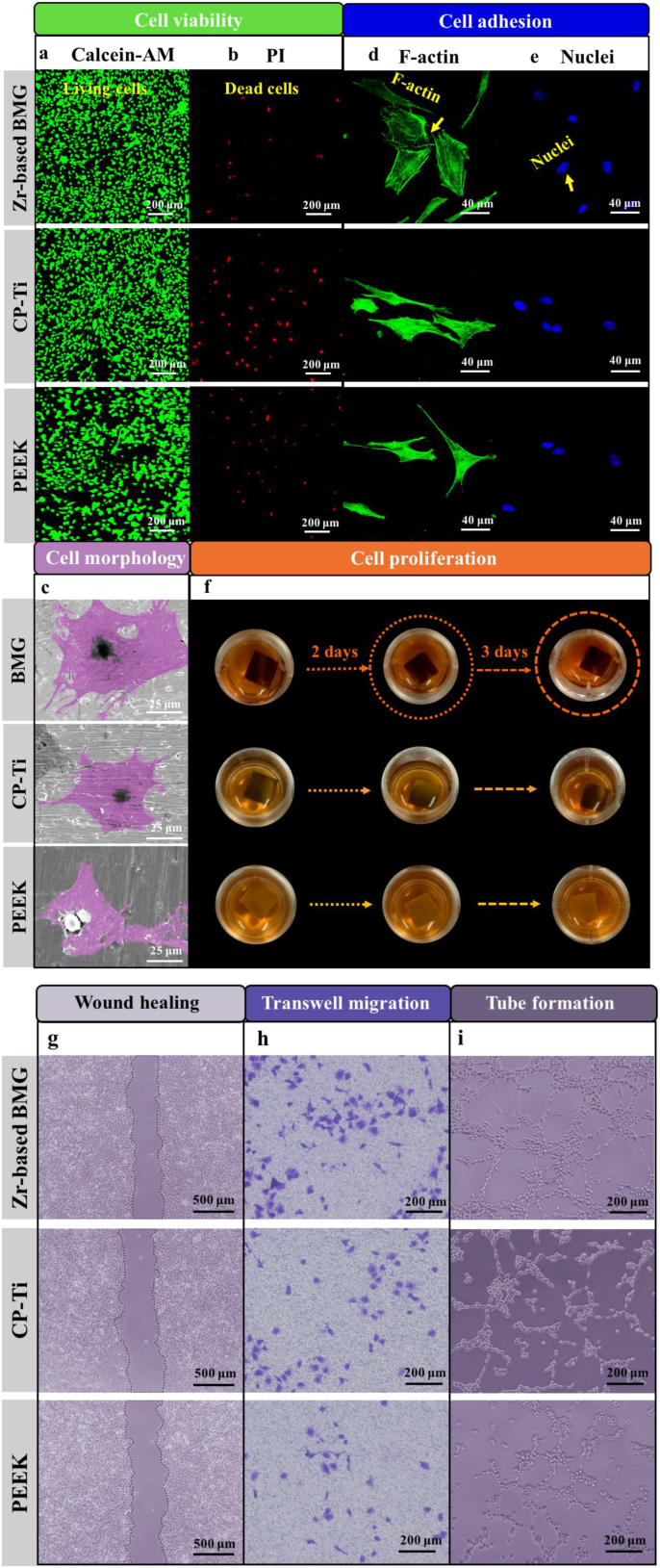
*In-vitro* cytocompatibility measurements with MC3T3-E1 pre-osteoblast for the Zr_61_Ti_2_Cu_25_Al_12_ BMG, CP-Ti, and PEEK. Cell viability was evaluated by a live/dead staining assay after 24 h of culturing. **a** The cells with green fluorescence are living cells. **b** The cells with red fluorescence are dead cells. PI refers to propidium iodide. **c** The morphology of cells. Cell adhesion analysis. Immunofluorescence imaging (F-actin marked by green – **d**, nuclei marked by blue – **e**). **f** Samples were immersed in CCK-8 solution for 1, 2, and 3 days. ***In-vitro* cell (HUVECs, Human umbilical endothelial cells) angiogenesis measurements of the Zr**_**61**_**Ti**_**2**_**Cu**_**25**_**Al**_**12**_**BMG, CP-Ti, and PEEK.** Wound healing assay: **g** The morphology of a wound after the migration of cells for 6 h and 24 h with each tested material. **h** The morphology of cells located on the lower surface of the Transwell pore. **i** Tube formation assay. The morphology of tubules formed with different materials.

The viability of MC3T3-E1 cells on the ZT1, CP-Ti, and PEEK is evaluated by a live/dead staining assay after 24-h of culturing. As shown in Fig. 3a – b, the cells with green fluorescence (marked by Calcein-AM) indicate the living cells, while the ones with red fluorescence (marked by PI) represent the dead cells. Compared with those of the CP-Ti and the PEEK, a denser population of living cells are found and only few, loosely scattered dead cells can be identified on the surface of the ZT1. The statistical analysis (Section IV of Supplementary Material (SM)) emphasizes that the ZT1 has obviously better cytocompatibility than the CP-Ti and the PEEK.

The morphologies of MC3T3-E1 pre-osteoblasts on the surface of various materials after culturing for 6 and 24 h are shown in Figs. S4 and Fig. 3c. After 24 h, more cells with extensively spreading and close contacting could be observed on the ZT1 (Fig. S4). The cells on the ZT1 are larger and more extensively spreading compared with those on the CP-Ti and the PEEK (Fig. 3c). Section IV of SM demonstrates a significant expansion of cells interacting with the ZT1 after increasing the culturing period to 24 h. The number of cells increases linearly with increasing the.

Culturing time, which may indicate indirectly that the ZT1 could support a cell proliferation more effectively as compared with other two materials. Significant differences among various materials take place after 24 h (Fig. S5), suggesting the ZT1 has a better, relatively bio-friendly interface for the cell attachment and growth. The analysis of morphology is consistent with our cell viability results shown in Fig. 3a and b, further supporting the *in-vitro* biocompatibility of the ZT1.

MC3T3-E1 pre-osteoblasts were cultured for 24 h on the surface of the ZT1, CP-Ti, and PEEK to investigate the cell adhesion behavior. The cells are fluorescently stained for F-actin (green, Fig. 3d) and nuclei (blue, Fig. 3e). The cells spread more extensively with clearer and better aligned actin filaments on the surface of the ZT1, whereas slimmer cell body morphology is observed on the surfaces of the CP-Ti and PEEK. The projected cell area (PCA) and an integrated optical density (IOD) (Section IV of SM) display significant differences in three materials. The morphology and histogram analysis reveal that the ZT1 can obviously promote the cell to spread, which result in the better cell adhesion behavior.

The cell proliferations on the ZT1, CP-Ti and PEEK after 1, 2 and 3 days are evaluated by CCK-8 assay shown in Fig. 3f. With increasing the duration of culturing, the color of the CCK-8 solution changes to a dark color for all of three materials, which indicates an obviously proliferating effect [23]. The solution is much darker in the case of the ZT1 as compared with those in the cases of the CP-Ti and PEEK at each time, demonstrating that the cells on the ZT1 are more viable. Based on the results of the statistical analysis of OD (optical density) in the CCK-8 assay (Section IV of SM), we can see that the ZT1 is non-cytotoxic and more strongly stimulated cell proliferation than the CP-Ti and PEEK.

The migrations of human umbilical endothelial cells (HUVECs) in the extracts of the ZT1, CP-Ti, and PEEK are analyzed by recording the movement of cells into the wound (wound healing assay). As shown in Fig. 3g, a more obviously wound narrowing occurs after 24 h for the ZT1 as compared with those of the CP-Ti and PEEK. The quantitative results (Fig. S5 in Section V of SM) displaying the percentage of wound healing of three materials suggest pronounced positive effects of the ZT1 on the cellular-migration repair response of HUVECs.

In order to measure the material-specific invasion capacity of HUVECs, the transwell migration assay was applied after 24-h co-culturing. As shown in Fig. 3h and Section V of SM, the number of HUVECs located on the low surface of the transwell pore is obviously higher in the ZT1 as compared to those in the CP-Ti and PEEK, demonstrating that the extract of the ZT1 can effectively increase the invasion capacity of HUVECs and stimulate cell migration.

The HUVECs angiogenesis potential of the ZT1, CP-Ti, and PEEK are evaluated by means of tube formation assay after 24-h co-culturing. As shown in Fig. 3i, several distinct tubules are generated in the group of the ZT1. Conversely, these tubules are rarely seen in the groups of the CP-Ti and PEEK. Quantitative analysis (Fig. S6) in Section V of SM demonstrates that the extract of the ZT1 effectively enhances an angiogenic ability.

Since our *in-vitro* measurements show that the ZT1 exhibits an apparently outstanding cytocompatibility and angiogenesis as compared with the CP-Ti and PEEK, *in-vivo* evaluations are subsequently employed in a rat model of femoral condylar insertion to further investigate the biocompatibility in a living body.

### 3.4 High-resolution micro-CT evaluation

**Fig. 4:**
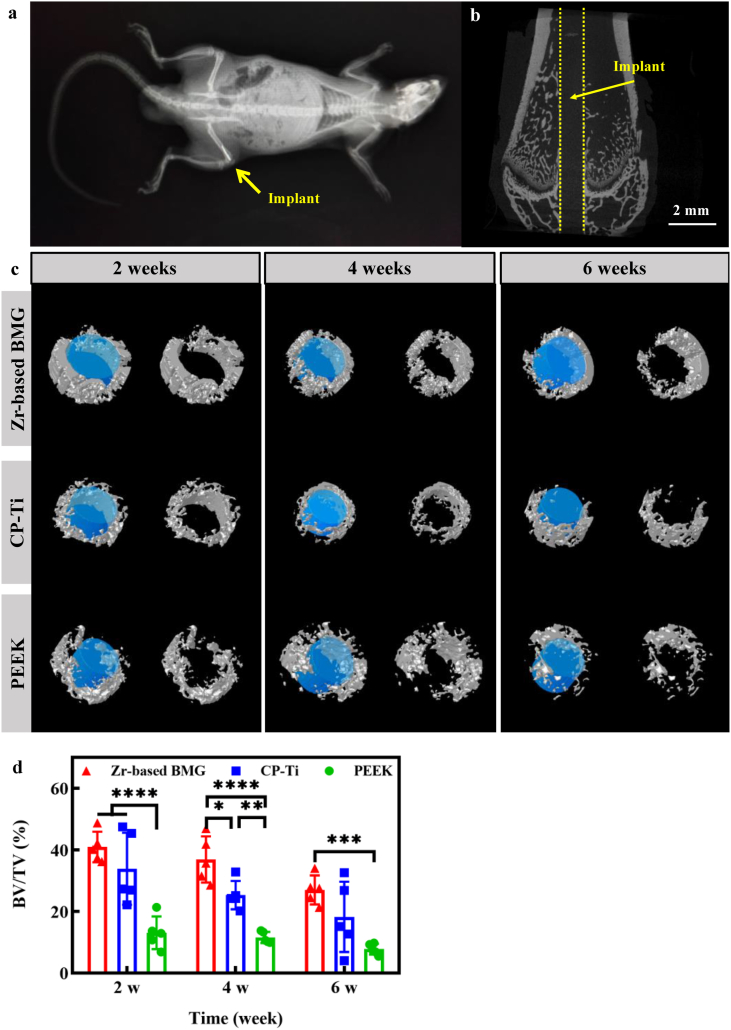
High-resolution micro-CT evaluation. a A radiographic image of the rat. **b** A reconstructed image of bone and the inserted implant. The yellow dot indicates the implant. **c** 3D reconstructions showing the morphology of the cancellous bone surrounding the implant at 2, 4, and 6 weeks after implanting the Zr_61_Ti_2_Cu_25_Al_12_ BMG, CT-Ti, and PEEK. **d** Inter-group variation in BV/TV (bone volume/total volume) at different time points. A *P* value < 0.05 was considered statistically significant, where 0.01 ≤ *P < 0.05, 0.001 ≤ **P < 0.01, 0.0001 ≤ ***P < 0.001, and ****P < 0.0001 shown in the histogram graphic.

The representative three-dimensional (3D) micro-CT reconstructions are shown in Fig. 4a – c and Fig. S7. The area in the radiographic image marked by the yellow dashed line indicates the position of the implant. The morphology of the reconstructed cancellous bone surrounding different implants after 2, 4, and 6 weeks (Fig. 4c) shows that the microarchitecture of the cancellous bone surrounding the ZT1 is fairly better than those around the CP-Ti and the PEEK. For a more quantitative assessment, the essential parameter of bone microarchitecture, the percentage of bone volume/total volume (BV/TV), is plotted in Fig. 4d. The BV/TV of the ZT1 after 2 weeks of implantation is particularly higher than that of the PEEK (****P < 0.0001). After 4 weeks, the ZT1 shows a higher BV/TV than the CP-Ti (*P = 0.03) and the PEEK (****P < 0.0001), while the CP-Ti and the PEEK significantly differ between each other (**P = 0.009). After 6 weeks, the ZT1 shows significantly higher BV/TV as compared with the PEEK (***P = 0.0003). It indicates higher *in-vivo* osteogenic potential for the ZT1 as compared with the other two materials.

### 3.5 Histological analysis

**Fig. 5:**
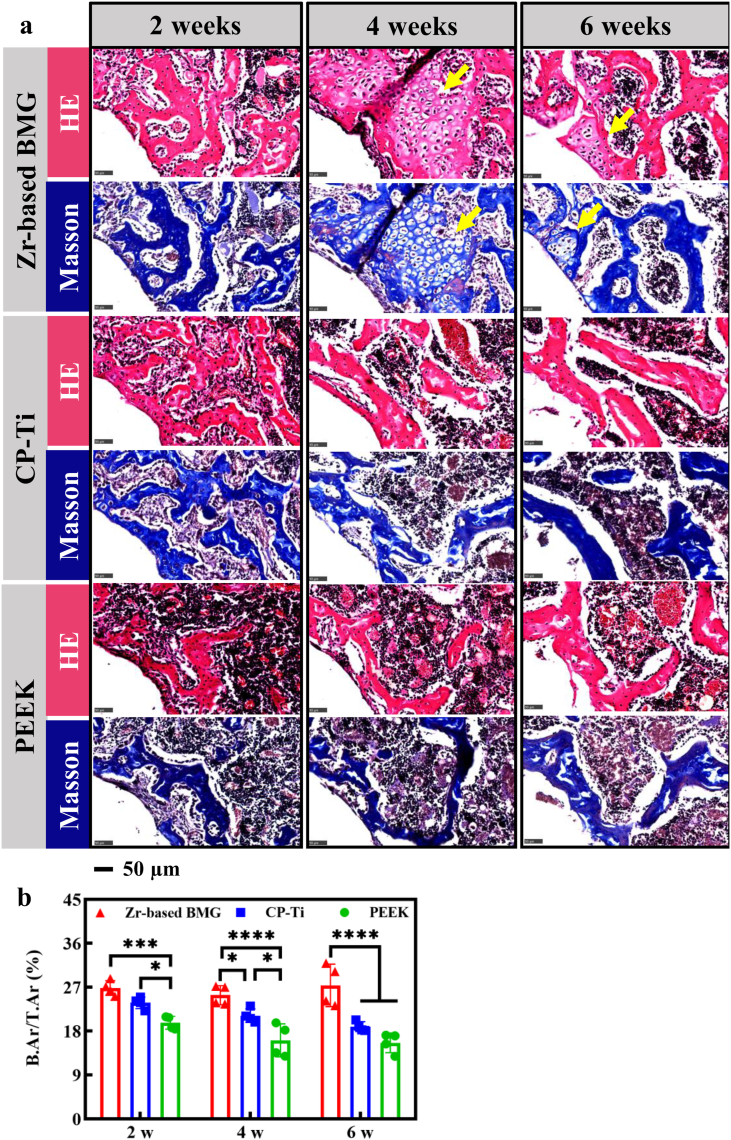
Histological analysis of the Zr_61_Ti_2_Cu_25_Al_12_ BMG, CP-Ti, and PEEK at different time points. a The HE and Masson's trichrome staining at magnifications of 40. The yellow arrows represented the active chondrogenesis observed around the Zr-based BMG. **b** The quantitative analysis of BA (bone area ratio). A *P* value < 0.05 was considered statistically significant, where 0.01 ≤ *P < 0.05, 0.001 ≤ **P < 0.01, 0.0001 ≤ ***P < 0.001, and ****P < 0.0001 shown in the histogram graphic.

To further analyze the osteogenic effects of the ZT1 on the cancellous bone, a histological analysis was conducted. Specifically, hematoxylin-eosin (HE) and Masson's trichrome staining were performed to assess the osteointegrations of the ZT1, CP-Ti, and PEEK after the implantation for 2, 4, and 6 weeks, respectively. The regions displayed with red and blue simultaneously can be regarded as bone tissue. As shown in Fig. 5a, a lamellar bone could be observed around the ZT1 after 2 weeks, where a trabeculae is formed as a ring around the circular edge of bone tunnel. In contrast, the trabeculae around the CP-Ti and the PEEK show more disorderly arrangement, extending radially outward from the bone tunnel. After 4 and 6 weeks, the typical lamellar bone is found around various materials. In addition, an active chondrogenesis is observed around the ZT1 (yellow arrows, Fig. 5a), but rarely seen around the CP-Ti and the PEEK. Since bone is a highly vascularized and heterogeneous tissue [24], the trabeculae well formed in the early stage provides an effective support for the hematopoietic tissue, which contributes additionally to the bone formation. A quantitative analysis of the bone area ratio (B.Ar/T.Ar), depicted in Fig. 5b, after 2 weeks, shows significant differences between the ZT1 vs. the PEEK (***P = 0.0004), and the CP-Ti vs. the PEEK (*P = 0.049). After the implantation of 4 and 6 weeks, the BA value of the ZT1 is significantly higher than those of the CP-Ti and the PEEK (*P =

0.04 for CP-Ti and ****P < 0.0001 for PEEK at 4 weeks; ****P < 0.0001 for CP-Ti and ****P < 0.0001 for PEEK at 6 weeks). Meanwhile, the CP-Ti differs from the PEEK after 4 weeks’ implantation (*P = 0.01). It is in line with the micro-CT findings and suggests that the ZT1 can stimulate active bone regeneration and early bone remodeling, resulting in a better osteointegration as compared with the CP-Ti and the PEEK.

### 3.6 Immunofluorescence analysis and imaging of blood vessels

**Fig. 6:**
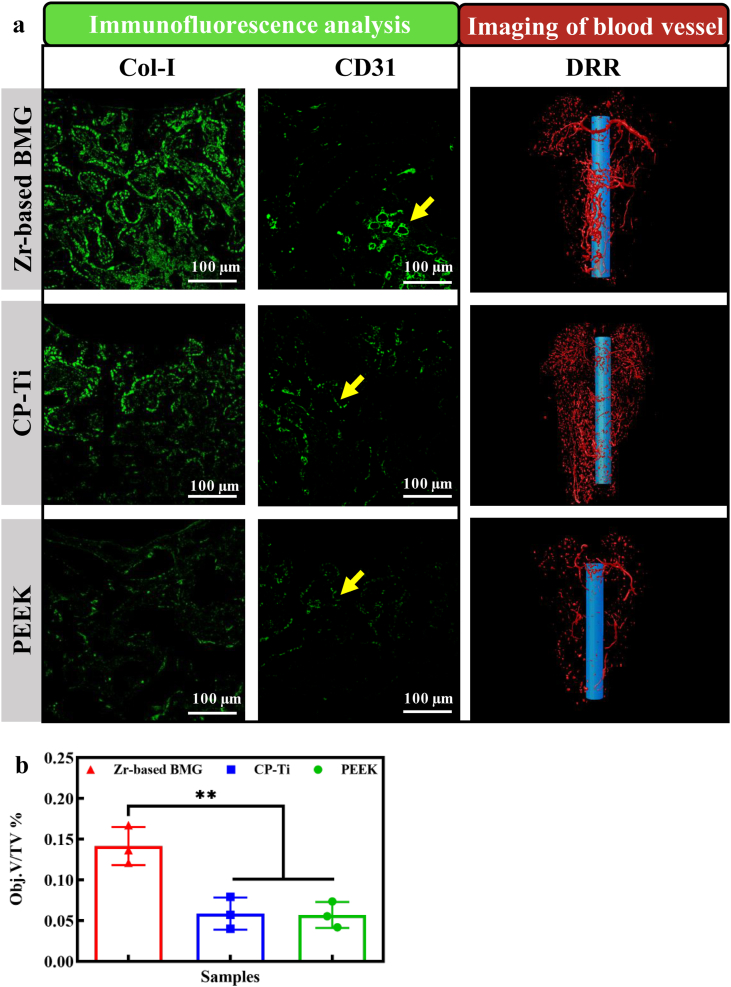
Immunofluorescence analysis and imaging of blood vessels of the Zr_61_Ti_2_Cu_25_Al_12_ BMG, CP-Ti, and PEEK. a The morphology of tissue section immunostained with Col-I. The morphology of tissue section immunostained with CD31. The digital reconstructed radiograph (DRR) by means of micro-angiography. **b** The vascular volume as a function of various materials. A *P* value < 0.05 was considered statistically significant, where 0.01 ≤ *P < 0.05, 0.001 ≤ **P < 0.01, 0.0001 ≤ ***P < 0.001, and ****P < 0.0001 shown in the histogram graphic-tibility of the ZT1 since no signs of systemic inflammation and adverse symptoms occur.

Based on the histological analysis, the lamellar bone is formed around the ZT1 after 2 weeks from the implantation. To further explore the mechanism at the protein level, the tissue sections are immunostained with Col-I and CD31. Col-I is an osteoblastic marker used to characterize early osteogenesis, while CD31 is a specific endothelial cell surface marker to characterize the angiogenesis. As shown in Fig. 6a, the expression levels of Col-I corresponding to the ZT1, the CP-Ti and the PEEK are strong, moderate, and minor, respectively. Regarding CD31, more immunofluorescence and more blood vessels are found in the ZT1. In contrast, for the CP-Ti and the PEEK, CD31 positive endothelial cells express lower immunofluorescence than the case in the ZT1. The statistical results depicted in Fig. S8 show that the density of Col-I and CD31 positive structures in the ZT1 group is significantly higher than those in the CP-Ti and the PEEK groups.

By means of micro-angiography imaging, the digital reconstructed radiographs (DRR) depict the formation of blood vessels around the samples (Fig. 6a). Main blood vessels are apparent around the ZT1, whereas around the CP-Ti and the PEEK mostly capillaries and only few main vascular channels are observed. In addition, the ZT1 shows higher vascular volume than the CP-Ti (**P = 0.005) and the PEEK (**P = 0.004) (Fig. 6b), which apparently promotes the formation of blood vessels, resulting in larger vascular volume postoperatively.

### 3.7 Elemental analysis at the implant-bone interface and hematological analysis

The accumulation of ions released to the surrounding bone was examined in the SEM-EPMA (Fig. S9). There is no pronounced accumulation of the ZT1 on the cross-sections of the dissected femora. Similar phenomena can be seen in the *in-vivo* ICP measurements (Fig. S10). The hematological analysis displays the routine blood and the biochemical parameters in the animals for the ZT1, the CP-Ti, and the PEEK after 2, 4, and 6 weeks (Figs. S11 – 12). All parameters are almost identical within the experimental error for the ZrT1, the CP-Ti, and the PEEK. Distinctions appear in some characteristics, e.g., red blood cell count (RBC), hematocrit (HCT), and hemoglobin (HB) etc.. However, they are not biologically meaningful as they are all within the typical range of biological variations in the living organisms. Nevertheless, the hematological results prove the *in-vivo* biocompa-

### 3.8 Gait analysis

**Fig. 7:**
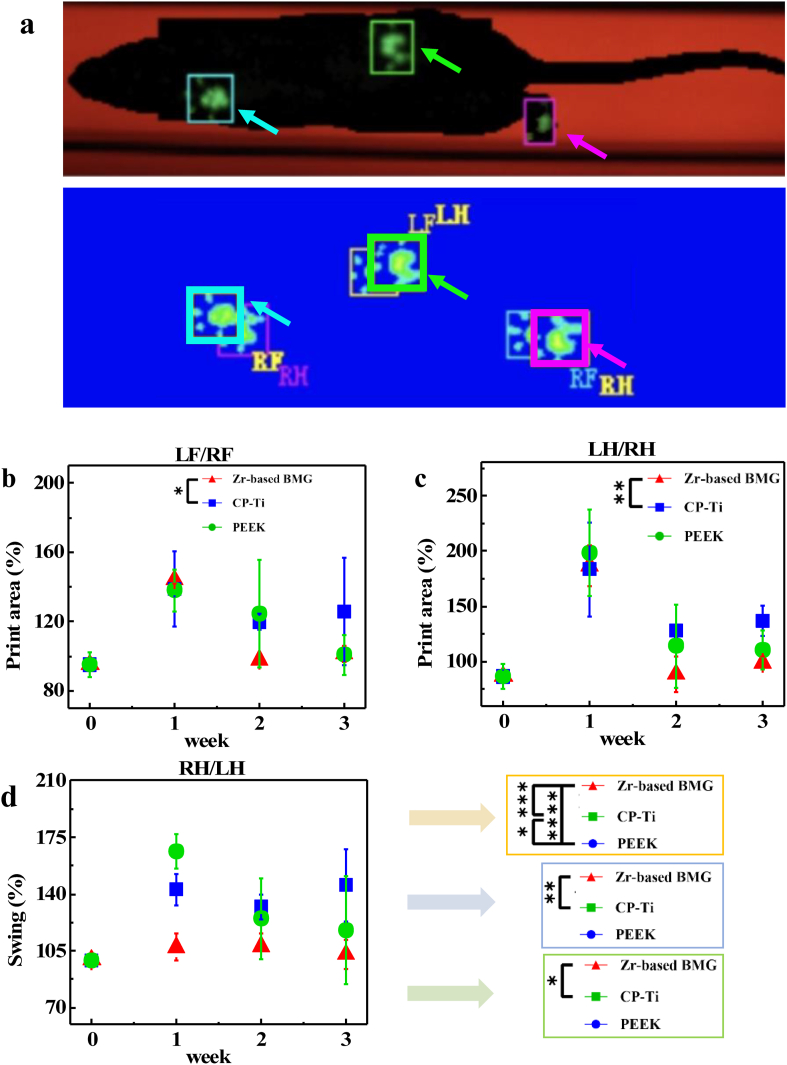
Gait analysis of the Zr_61_Ti_2_Cu_25_Al_12_ BMG, CP-Ti, and PEEK at different time points. a The screenshot of the experiential process from the CatWalk automated gait analysis system, the cat-walking rat, and the corresponding footprint. **b** The print area of LF/RF. **c** The print area of LH/RH. **d** The swing duration of RH/LH. A *P* value < 0.05 was considered statistically significant, where 0.01 ≤ *P < 0.05, 0.001 ≤ **P < 0.01, 0.0001 ≤ ***P < 0.001, and ****P < 0.0001 shown in the histogram graphic.

Apart from the tissue and protein analysis, the measurement of pain is regarded as one of the major outcome dynamic methods, which is available in several animals [25]. A gait analysis aims to estimate the acute effect of various implants in the living body by measuring pain levels (Video. 1). We conducted the gait analysis preoperatively as well as 1, 2, and 3 weeks after the implantation in rats. Since the materials were implanted into the right hind (RH) leg, more painful feeling is expected to occur in the RH rather than in other extremities. Consequently, the values of print intensity and area from the RH must be smaller and the corresponding paw elevation time must be longer, which are directly proportional to the intensity of pain, whereas the opposite would be expected for the left front (LF) leg. Fig. 7a displays the screenshot from the software, giving an insight into the process of the measurement. The print area of LF/RF (Fig. 7b) of rats with an implant made of the ZT1 is lower than that of the CP-Ti after 2 weeks implantation (*P = 0.01). Significant difference is observed from the print area of LH/RH (Fig. 7c) between the ZT1 and the CP-Ti for 2 and 3 weeks (**P = 0.009 and **P = 0.004, respectively). The swing duration of RH/LH is shown in Fig. 7d. One week after surgery, significant differences are observed between various materials (the ZT1 vs. the CP-Ti: ***P = 0.0007, the ZT1 vs. the PEEK: ****P < 0.0001, the CP-Ti vs. the PEEK: *P = 0.02). After implantation for two weeks, the swing time of RH/LH in the rats with the ZT1 implants is lower than those in the rats with other two materials. Moreover, this tendency still exists after 3 weeks (*P = 0.02). The rats with the ZT1 implant shows the lowest values than those of other two materials. The avoidance mechanism of mechanical allodynia can be achieved by increasing paw-elevation time [25]. Therefore, a longer swing time indicates a stronger pain feeling. The dynamic gait analysis demonstrates that the ZT1 tends to a lower pain level during recovery, avoiding suffering from the avoidance mechanism, which can reduce the sensitivity of mechanical allodynia induced by the implants.

## 4 Discussion

The surface properties, such as surface chemistry, porosity, roughness, wettability and oxide film, are known as important factors affecting the bio-corrosion behavior and biocompatibility (e.g., cellular adhesion, proliferation, and differentiation) [[26], [27], [28]]. In the present study, the BMG exhibits a compact structure. Thus, the porosity on the surface of the BMG does not significantly influence the results [26]. As shown in Fig. S2b, the initial surface roughness does not notably differ within the ZT1, the CP-Ti, and the PEEK. Besides roughness, hydrophilicity and its related surface energy are equally important [29]. Compared with those of the CP-Ti and the PEEK, the wettability of the ZT1 points.

Towards more hydrophilic behavior, which is a preferred property in clinic [30]. Based on the measured contacted angle, *θ*, the adhesion tension, *τ*, can be approached by τ=γcosθ, where *γ* = 72.8 dyn/cm for water [31]. Accordingly, a smaller contacted angle will lead to a larger adhesion tension, i.e., the

Higher surface energy, resulting in more rapid cell activation and differentiation [29]. Moreover, the enhanced interaction between the implant surface and the biological environment can achieve the promising bone regeneration, providing a reliable interpretation of the better osseointegration for the ZT1 at the early stage [32]. It should be noted that the wetting behaviors in air and humidity conditions are significantly different [26]. In the present study, the hydrophilicity measurement was carried out at the temperature of 25 °C and the relative humidity of 41%.

**Fig. 8:**
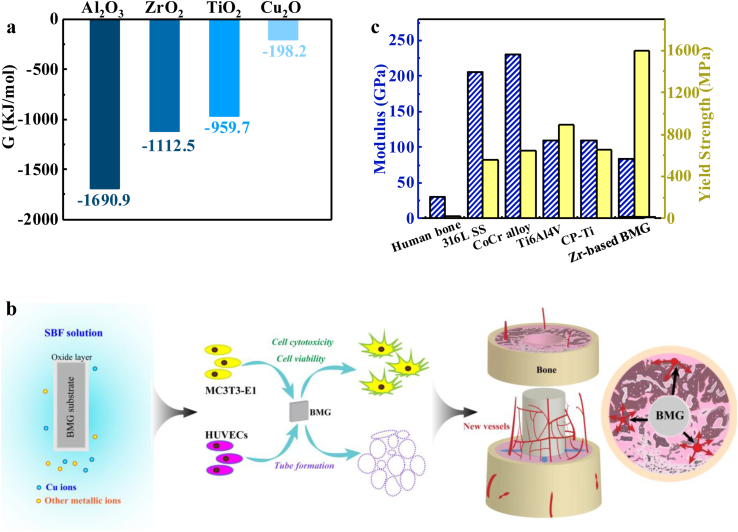
(a) The thermodynamic quantity. The Gibbs free energy for Al_2_O_3_, ZrO_2_, TiO_2_, and Cu_2_O at room temperature. (b) The schematic diagram of osteogenesis and angiogenesis of the ZT1 that can stimulate an early blood vessel formation (as the black arrows indicated). This is benefit to a bone regeneration after implanting (as the red arrows indicated). (c) Mechanical properties. The modulus, strain limit, and strength of cortical bone, typical implant materials (316L SS, CoCr alloy, Ti6Al4V, CP-Ti), and the ZT1.

In addition, an oxide film formed on the surface is another essential aspect. Compared with the single oxide compound, TiO_2_, formed in the CP-Ti (Fig. S3k), the constituents of the oxide film in the ZT1 are multiplied. As shown in Fig. 8a, the Gibbs free energy of forming of Al_2_O_3_, ZrO_2_, TiO_2_, and Cu_2_O at room temperature is −1690.9, −1112.5, −959.7, and −198.2 kJ/mol, respectively. According to the Ellingham-Richardson diagrams, the more negative the standard free energy change of formation of oxide is, the more stable the corresponding compound is [33]. Therefore, the major oxide component, ZrO_2_, is more structurally dense. Meanwhile, it is chemically stable [34], as well as no cytotoxic [35], which is largely responsible for the positive behavior of the ZT1. The coexistence of embedded *i*-oxide (*i* = Ti, Al) appears to enhance the property of the passive film, which leads to a relatively high corrosion resistance [36,37]. Meanwhile, some studies have clarified that the effect of Cu on the corrosion resistance is somewhat analog to that of noble metals, being supposed to achieve a much nobler potential for a given alloy [36]. Although the XPS analysis reveal the presence of Cu_2_O on the surface, it is unstable and easily reduced. The corresponding essential reaction process of Cu in SBF could be inferred from these equations:$2\text{Cu}+1/2{\text{O}}_{2}\to {\text{Cu}}_{2}\text{O}$, $\text{Cu}+n{\text{H}}_{2}\text{O}\to {\text{Cu}}_{m}^{0}{\left({\text{OH}}^{-}\right)}_{n}+n{\text{H}}^{+}$; ${\text{Cu}}_{m}^{0}{\left({\text{OH}}^{-}\right)}_{n}+\left(n+m\right){\text{H}}^{+}+\text{m}/2{\text{O}}_{2}\to m{\text{Cu}}^{2+}+\left(n+m\right){\text{H}}_{2}\text{O}$ [38]. Therefore, Cu can survive in an ionized form in the biological environment. The effective oxide films suggest that the ZT1 can be a reliable candidate for the biological applications, giving its ability to prolong implant life.

The existence of a surface oxide film can form a protective barrier to improve the corrosion resistance and simultaneously act as a medium to enhance biocompatibility. However, the film can be damaged in the living body during wear and fretting [39]. In this case, some products, e.g., wear debris, ions, and oxides, are likely to be released into the surrounding environment through various mechanisms [37], which can affect both the safety of the living body and the biocompatibility of the material. In general, the released ions are regarded as the most crucial potentiality, essentially affecting the physiological environment [35]. Therefore, avoiding the absence of toxic elements, such as Ni, Cr, Co, Be, and Nb, is principally necessary to prevent inflammatory reactions [37]. Abnormal ionic accumulation is also not allowed, which markedly changes depending on the composition and (micro)structure of the alloy, the strength of the metal-oxide bond, and the thicknesses of the oxide films, as well as complex environmental factors [40]. The Zr_61_Ti_2_Cu_25_Al_12_ BMG shows excellent biocompatibility and promoted osteogenesis and angiogenesis owing to the physiological roles resulted from the different constituent elements.

Zr and Ti are regarded as inert elements, whose toxicity are acknowledged to be minimal due to the lack of combination with biomolecules. Their alloys are nontoxic, accompanied with good biocompatibility. For Zr, its ceramics and coatings exhibit excellent osteointegration [35]. For Ti, its releasing concentration from the ZT1 during the ICP measurements is basically as same as that from the CP-Ti. There is also no abnormal ionic accumulation due to the formation of its stable oxide [35], TiO_2_, as shown in the interface analysis (Fig. S9) and *in-vivo* ICP (Fig. S10).

Al is a potentially necrotic element, which might be related to Alzheimer's-type senile dementia [41]. However, Al-ions only show the cytotoxicity when their concentrations exceed 200 ppb [42]. In the present study, Al is mostly detected in the form of Al_2_O_3_, which is the stable oxide [35]. Therefore, the concentration of released Al ions is far below the level that can cause cytotoxicity. Moreover, no inflammatory reactions occurred in the *in-vitro* and *in-vivo* experiments, indicating that presence of Al in the Zr_61_Ti_2_Cu_25_Al_12_ BMG is within a safe range. In addition, Gillian et al. [41] have found that Al may accumulate in the brains of individuals with defective transferrin. Without this defective transferrin, the neurotoxic effects of aluminum in Alzheimer disease and Down syndrome can be avoided.

Although a Cu ion is necessary for human body, its excessive concentration leads to a biological toxicity [43]. In the present study, the concentration of Cu ion released from the ZT1 is 33.8 ppb after 1 week of immersion, which is far below the threshold level suggested by the World Health Organization (WHO) [44]. After 2–6 weeks, no abnormal accumulation of Cu ions can be seen, indicating its safety in the ZT1. Table 1 summarizes the released concentration of Cu ion found from previously investigated BMGs, which displays a comparable magnitude. Specific differences between them is probably due to lots of aspects, e.g., the composition of systems, immersing solutions, and the immersing duration.

**Table 1:** The concentration of Cu ions released from Zr-, Ti-, Sr-, Mg-based BMGs, Cu-doped 45S5 Bioglass®, and traditional Zr-1Cu alloy at various solutions after different immersing durations.

BMG	Concentration (ppb)	Days	Solution	Ref.
Zr_61_Ti_2_Cu_25_Al_12_	34	7	SBF	This work
Zr_61_Ti_2_Cu_25_Al_12_	45	14	SBF	This work
Zr_61_Ti_2_Cu_25_Al_12_	38	28	SBF	This work
Zr_61_Ti_2_Cu_25_Al_12_	30	42	SBF	This work
Zr_60.14_Cu_22.31_Fe_4.85_Al_9.7_Ag_3_	168.6	30	ABF	[3]
Zr_55_Al_10_Ni_5_Cu_30_	≈100	1	PBS	[45]
(Zr_55_Al_10_Ni_5_Cu_30_)_99_Y_1_	≈70	1	PBS	[45]
Zr_55_Al_10_Ni_5_Cu_30_	≈500	7	PBS	[45]
(Zr_55_Al_10_Ni_5_Cu_30_)_99_Y_1_	≈100	7	PBS	[45]
Zr_62.5_Cu_22.5_Fe_5_Al_10_	161	7	SBP	[46]
Zr_58_Cu_22_Fe_8_Al_12_	622	2	PBS	[43]
Zr_58.8_Cu_15.6_Ni_10.3_Nb_2.8_	868	2	PBS	[43]
Zr_50_Cu_43_Al_7_	33	1	DMEM	[47]
Ti_40_Cu_38_Zr_10_Pd_12_	≈39	7	MEMα	[10]
Ti_40_Cu_38_Zr_10_Pd_12_	≈37	21	MEMα	[10]
Ti_41.5_Zr_2.5_Hf_5_Cu_37.5_Ni_7.5_Si_1_Sn_5_	2300	3	DMEM	[48]
Sr_40_Mg_20_Zn_15_Yb_20_Cu_5_	150	3	Hank's SBF	[49]
Mg_65_Ag_12.5_Cu_12.5_Y_10_	360	90 min	H_2_SO_4_	[50]
Cu-doped 45S5 Bioglass®	14000	3	DMEM	[51]
Zr-1Cu alloy	<1	30	Hank's SBF	[36]

ABF: Artificial body fluid.

PBS: Phosphate buffered saline.

SBP: Simulated blood plasma.

DMEM: Dulbecco's modified Eagle's medium.

MEMα: Minimum essential medium α

The *in-vitro* ICP analysis suggests that the most evident and significant difference in the released ions between the ZT1 and CP-Ti can be characterized as the Cu ionic concentration, which may influence the surrounding bone and tissue. However, there is no obvious distinction in the Cu ionic concentration from the *in-vivo* ICP result, probably due to two reasons, one is likely owing to the locality of ionic release and reactions, and another one may be the effect of the regulatory mechanism. This generally operates whenever abnormality occurs. The living body instinctively metabolizes in order to keep balance and avoid induced systemic changes in itself, consequently, resulting in undetected differences *in-vivo*. Cu is reported to be a key element affecting blood vessel growth, stimulating angiogenesis at the molecular level [52]. The angiogenic activity of Cu is attributed to an up-regulation of hypoxia inducible factor (HIF)-1α [53], VEGF [54] as well as endothelial nitric oxide synthase expression [55], which has been proved by Cu-droped 45S5 bioglass [51]. Li et al. [19] have revealed that the Zr_61_Ti_2_Cu_25_Al_12_ BMG significantly promotes the gene expression of VEGF (Vascular endothelial growth factor), contributing the regulation of vasculogenesis. It is consistent with our *in-vitro* experiments with HUVECs (Fig. 3g – i), *in-vivo* immunofluorescence tests, and DRR analysis (Fig. 6). Therefore, we rationally hypothesize that the “noble character” of Cu and its unstable oxide achieve an optimal concentration in the environment, playing a positive role in angiogenic ability.

The immunofluorescence analysis renders a realistic interpretation of the findings from micro-CT, and histological analysis from the perspective of proteins and molecular level. The bone is formed by two independent mechanisms, viz., intramembranous and endochondral ossification [56]. The endochondral ossification can account for the development in most bones, which includes a two-stage mechanism, i.e., chondrocytes form a matrix template, and growth plate chondrocytes undergo well-ordered and controlled phases of cell proliferation, maturation, and death. This unique differentiation process is followed by a blood vessel invasion and a replacement of the cartilaginous matrix with bone [57]. Therefore, the blood vessel invasion is a critical event in the replacement of cartilage by bone and in the formation of bone marrow cavity [24]. In the current study, the biological role of blood vessels in bone is far beyond a mere source of nutrients [24]. The achievement of bone regeneration centers on the dominant role of vascularization since the effective delivery of nutrients, minerals, growth factors, and oxygen for tissue restoration [58]. Moreover, signals emanating from vascular cells accelerates the osteogenesis, and increases the number of blood vessels, which introduces more osteoblast progenitors to further increase the bone formation [24], and thereby plays a critical role in skeletal development and repair. It reflects the enhanced angiogenic potential of the Zr_61_Ti_2_Cu_25_Al_12_ BMG, which achieves earlier promotion of formation of blood vessels, eventually favoring osteointegration (Fig. 8b).

Biomechanical properties are fundamental in orthopedic applications in order to adapt to the requirements of hard tissue replacement. The most vital evaluation indicator, i.e., stress shielding, must be significantly taken into consideration to avoid loosening and failure of implants. Specifically, the Young's modulus of the implant material should ideally be similar to that of the bone. Meanwhile, a superior strength together with a high hardness can give rise to a better wear resistance, which also improves the capacity of the material for load-bearing applications [59]. Furthermore, the recovery of strain of biological materials is larger than 2% after deformation [60], achieving the ability to flex elastically with the natural bending of the bone. As Fig. 8b illustrated, compared to the mechanical properties of human bone [61], most of the implants (316L SS, CoCr alloy, Ti6Al4V, and CP-Ti) follow the requirements for a suitable Young's modulus and high strength, and large strain limit [62]. In the current study, compared with the alloys previously used, the ZT1 has a more suitable Young's modulus (83 GPa) accompanying with significantly high yield strength (1688 MPa) [20]. The Young's modulus of bone is in a wide range from 10 to 40 GPa [4,14]. Thus. the stress-shielding phenomenon cannot be avoided completely in the present study. For the alloy composition of ZT1, the addition of Ti and the lower atomic concentration of Cu can achieve a lower Young's modulus, as compared to the Young's moduli of a Zr_60_Cu_25_Al_12_ BMG (84 GPa) and a Zr_48_Cu_45_Al_7_ BMG (90 GPa) [18]. To further decrease the moduli of alloys for satisfying the bio-application, more work concerning composition design are required. Based on the current research, the excellent biomechanical properties make the ZT1 to be a reliable material for medical applications, ensuring improved loading condition of the surrounding bone and achieving a long-term servicing period after surgery.

## 5 Conclusions

In summary, the Zr_61_Ti_2_Cu_25_Al_12_ BMG is prepared and subjected to systematic bio-corrosion and biocompatibility analyses and compared with the benchmark materials of the CP-Ti and the PEEK. The chemical measurements indicate that the corrosion resistance of the BMG is superior to the CP-Ti owing to the effective passivation coming from multiple oxide films at the glass's surface. Regarding the *in-vitro* experiments, the BMG shows a relatively bio-friendly interface for MC3T3-E1 pre-osteoblast attachment and growth, and stimulated cell proliferation. It also displays positive effects on HUVEC cellular migration repair response and enhanced invasion capacity. The *in-vivo* radiographic evaluation and histological analysis demonstrate the BMG's positive effects on promoting growth of cancellous bone. Accompanying with a better osseointegration, better angiogenesis, i.e., earlier promotion of the formation of blood vessels in case of the BMG, is also detected by immunofluorescence analysis and DRR. The hydrophilic characteristics of the 10.13039/501100003107BMG support better osseointegration at the early stage after implantation. The moderate concentration of released Cu ion influences the growth of blood vessel, achieving the interplay between angiogenesis and osteogenesis. In addition, the *in-vivo* ICP results and hematological analysis do not provide any hints regarding abnormal ion accumulation and inflammatory reactions in the living organisms. Dynamic observation of gait shows that rats that are implanted the BMG tend to have a lower pain level during recovery as compared to those who received the CP-Ti or PEEK implant. Our work strongly suggests that the Zr_61_Ti_2_Cu_25_Al_12_ BMG is a suitable candidate for biological use.

## Author contributions

**Kang Sun**, **Rao Fu**, **Xing-Wang Liu**, and **Gang Wang** proposed idea and wrote manuscript, **Kang Sun**, **Li-Ming Xu**, and **Xing-Wang Liu** carried out the experiments, **Qi-Jie Zhai** and **Simon Pauly** revised and corrected the manuscript, **Kang Sun**, **Xing-Wang Liu**, and **Gang Wang**, **Li-Ming Xu** analyzed the date. All authors discussed the results and reviewed the manuscript.

## CRediT authorship contribution statement

**K. Sun:** Formal analysis, Writing – review & editing, proposed idea and wrote manuscript, carried out the experiments, analyzed the date. All authors discussed the results and reviewed the manuscript. **R. Fu:** Writing – review & editing, proposed idea and wrote manuscript, All authors discussed the results and reviewed the manuscript. **X.W. Liu:** Formal analysis, Writing – review & editing, Writing – original draft, proposed idea and wrote manuscript, carried out the experiments, analyzed the date. All authors discussed the results and reviewed the manuscript. **L.M. Xu:** Formal analysis, Writing – review & editing, carried out the experiments, analyzed the date. All authors discussed the results and reviewed the manuscript. **G. Wang:** Formal analysis, Writing – review & editing, Writing – original draft, proposed idea and wrote manuscript, analyzed the date. All authors discussed the results and reviewed the manuscript. **S.Y. Chen:** Writing – review & editing, All authors discussed the results and reviewed the manuscript. **Q.J. Zhai:** Writing – review & editing, revised and corrected the manuscript, All authors discussed the results and reviewed the manuscript. **S. Pauly:** Writing – review & editing, revised and corrected the manuscript, All authors discussed the results and reviewed the manuscript.

## Declaration of competing interest

The authors declare that they have no known competing financial interests or personal relationships that could have appeared to influence the work reported in this paper.
