# Supplementary material for: Osteogenesis and angiogenesis of a bulk metallic glass for biomedical implants
Source: Bioact Mater. 2021 Jun 20;8:253–66. doi: 10.1016/j.bioactmat.2021.06.018 (PMC8424448; doi:10.1016/j.bioactmat.2021.06.018)
Supplement: Multimedia component 1 [file mmc1.docx]

**Osteogenesis and angiogenesis of a bulk metallic glass for biomedical implants**

K. Sun^1,#^, R. Fu^2,#^, X.W. Liu^3^, L.M. Xu^1^, G. Wang^1,*^, S.Y. Chen^3,*^, Q.J. Zhai^1^, S. Pauly^4^

^1^ Institute of Materials, Shanghai University, Shanghai 200444, China

^2^ Department of Neurology, Tongren Hospital, School of Medicine, Shanghai Jiao Tong University, Shanghai 200336, China

^3^ Sports Medicine Department of Huashan Hospital, Fudan University, Shanghai 200040, China

^4^ University of Applied Sciences Aschaffenburg, Würzburger Straße 45, D-63743 Aschaffenburg, Germany

^#^First authors

*Corresponding author: [g.wang@i.shu.edu.cn](mailto:g.wang@i.shu.edu.cn) and [cshiyi@163.com](mailto:cshiyi@163.com)

**I. *In-vivo* evaluation**

The *in-vivo* surgery process is shown in Fig. S1. A medial parapatellar arthrotomy was performed to expose the right intercondylar fossa, which was the deep notch between the medial and lateral femoral condyle. A 10-mm long bone tunnel was drilled through the intercondylar fossa with a Ф 1.2 mm Kirschner wire. Subsequently, the sample was inserted into the tunnel. After rinsing the joint with PBS, the fascia and skin were closed in layers. The rats were euthanized at 2, 4, and 6 weeks after surgery by the administration of an overdose of chloral hydrate.

**Fig. S1. The *in-vivo* surgery process.** **a** A medial parapatellar arthrotomy was performed to expose the right intercondylar fossa. **b** A 10-mm long bone tunnel was drilled through the intercondylar fossa with a Ф 1.2 mm Kirschner wire. **c** The morphology of the bone tunnel. **d** The sample was inserted into the tunnel. **e** The sample had been inserted. **f** After rinsing the joint with PBS, the fascia and skin were closed in layers. The rats were euthanized at 2, 4, and 6 weeks after surgery by the administration of an overdose of chloral hydrate.

**II. Microstructural and hydrophilic characterization.**

Figure S2a shows the surface morphology observed by SEM with a system build-in surface analysis program, giving roughness values, *R_a_*, of 647 ± 15, 644 ± 26 and 642 ± 10 nm for the Zr-based BMG, CP-Ti, and PEEK, respectively (Fig. S2b). There are no significant differences between them since *P* values for the Zr-based BMG vs. CP-Ti, the Zr-based BMG vs. PEEK, and CP-Ti vs. PEEK is 0.97, 0.91, and 0.98. The hydrophilicity of various materials was estimated based on contact-angle measurements of a sessile drop of deionized water placed on the material (Fig. S2c).

**Fig. S2. Microstructural and hydrophilic characterization of the investigated materials. a** The surface morphology of materials obtained by SEM. The roughness values are given by the insert images from a system build-in surface program. **b** The roughness values of the Zr_61_Ti_2_Cu_25_Al_12_ BMG, CP-Ti, and PEEK. **c** Hydrophilic characterization of the Zr_61_Ti_2_Cu_25_Al_12_ BMG, CP-Ti, and PEEK.

**III. Release of metal ions and corrosion behavior**

Figure S3a shows the results of ICP-MS, i.e. the cumulative ionic concentration released in SBF solution (Simulated body fluid: 8.0 g/L NaCl, 0.4 g\L KCl, 0.35 g/L NaHCO_3_, 0.14 g/L CaCl_2_, 1.0 g/L C_6_H_6_O_6_ (glucose), 0.2 g/L MgSO_4_·H_2_O, 0.1 g/L KH_2_PO_4_·H_2_O and 0.06 g/L Na_2_HPO_4_·H_2_O; pH = 7.4; at the human body temperature: 37 °C [1]) respectively from the Zr_61_Ti_2_Cu_25_Al_12_ BMG and CP-Ti as a function of immersing time. As depicted, Zr, Cu, Al and Ti ions were detected for Zr-based BMG. After 1 week’s accumulation, the concentration of Zr, Al and Ti ions are 1.1, 3.3 and 5.9 ppb, respectively. By contrast, the ion concentration of Cu is extremely higher with the value of 33.8 ppb, occupying the percentage of 76.6% of all elements. After 2 to 6 weeks, the ion concentration of Zr is around 1.1, 1.0 and 2.8 ppb, respectively. For Al, it is at the level of 1.4, 3.2 and 1.9 ppb. And for Ti, it is 4.8, 5.1 and 4.3 ppb. The releasing of Cu ion exhibits consistently the most with the value of 44.8, 38.4 and 29.7 ppb. There are no significant changes of the cumulative ion concentration at different period, demonstrating metal ions were generally releasing to the SBF solution at the initial stage of immersing. It is in agreement with previous study, suggesting Cu ion appears to approach the saturation at longer dissolution times [2]. Meanwhile, the concentration of Cu ion released to the solution is 6 to 30 times at each point compared with other elements in the Zr-based BMG. With regard to CP-Ti, the concentration of released Ti ion is 8.2, 9.5, 9 and 10.4 ppb at 1, 2, 4 and 6 weeks, respectively. Similar to the Zr-based BMG, no obvious changes can be discovered at different immersing period. Simultaneously, the total release of ions from the Zr-based BMG after 1 week’s immersion is 44.1 ppb, approximately 5 times higher than CP-Ti with 8.2 ppb, which is attributed to the high concentration of Cu.

**Fig. S3. Corrosion behavior of the Zr_61_Ti_2_Cu_25_Al_12_ BMG and CP-Ti.** **a** Release of metal ions (Zr, Cu, Al and Ti). **b** Tafel curves from the electrochemical corrosion test and the corresponding characteristic parameters, i.e. the corrosion voltage, *E_corr_* and the corrosion current, *I_corr_*. The counts as a function of binding energy for different elements on the surface and internal layers investigated by XPS. **c** For Zr on the surface. **d** For Zr 3*d*. **e** For Cu on the surface. **f** For Cu 2*p*. **g** For Al on the surface. **h** For Al 2*p*. **i** For Ti from Zr-based BMG. **j** For Ti 2*p* from the Zr-based BMG. **k** For Ti from CP-Ti. **l** For Ti 2*p* from CP-Ti.

Figure S3b shows the Tafel curves of the Zr_61_Ti_2_Cu_25_Al_12_ BMG and CP-Ti from electrochemical corrosion tests, giving the values of characteristic parameters, i.e. the corrosion voltage, *E_corr_* and the corrosion current, *I_corr_*. In general, the higher value of *E_corr_* accompanying with the lower value of *I_corr_* demonstrates a better corrosion resistance. As it shown, the *E_corr_* values of Zr-based BMG and CP-Ti are -0.41 mV and -0.5 mV, respectively. *I_corr_* values of both materials are 2.659 ×10^-6^ A/cm^-2^ and 1.62×10^-5^ A/cm^-2^. It indicates the Zr_61_Ti_2_Cu_25_Al_12_ BMG has better corrosion resistance than CP-Ti in physiological solutions, which is attributed to the formation of protective oxide film [3].

In order to explore the specific products on the surface and internal layer, Figs. S3c-l illustrate the chemical state of elements with 50 seconds’ interval after etching electrochemical corrosion investigated by XPS. For the Zr_61_Ti_2_Cu_25_Al_12_ BMG, the Zr 3*d* spectrum consists of two peaks (Fig. S3c), at approximately 182.41 and 184.8 eV. They are corresponding to Zr 3*d*5/2 and Zr 3*d*3/2 electrons of the Zr^4+^ oxide state [4], indicating the oxide produce is ZrO_2_. The chemical state of Zr on various layer was explored with etching, which is shown in Fig. S3d. The curves after 200 s do not plot obvious differences, and the relative counts’ values suggest the chemical state of Zr *3d* has become 0. Cu 2*p* spectrum at 0s exhibits two peaks at 932.82 and 952.63 eV (Fig. S3e), likely belonging to Cu 2*p*3/2 and Cu 2*p*1/2, respectively [5]. The bonding energies of metallic Cu state is in the range of 932.2 – 933.1 eV, while for Cu_2_O, it is within 932 – 932.8 eV [4]. Therefore, the corresponding spectrum on the surface represents the presence of metallic Cu state and the Cu_2_O compound. The peak position of two peaks (the bonding energy of Cu and Cu_2_O are 952.56 eV and 952.5 eV, respectively) are slightly shifted to higher values of 50s’ spectrum (Fig. S3f), and they do not change after that, it indicates the metallic Cu state inside. In addition, the chemical state of Al 2*p* and Ti 2*p* in the Zr-based BMG on the surface and different layers are provided in Figs. S3g - j. The Al 2*p* spectrum shows two spectras, peaking at 74.42 and 76.73 eV, corresponding to Al 2*p*3/2 electrons from the Al^3+^ oxide state [6]. For Ti 2*p*, it only has one peak, at 458.61 eV, corresponding to Ti 2*p* from Ti^4+^ oxide state. It suggests the corresponding oxide produce for Al is Al_2_O_3_, and for Ti is TiO_2_. The changes of Al and Ti curves are suspending after 150 s, early than Zr with 200 s. It might be owing to their small amount of atomic percentage or the difficulties of their oxide film’s formation. Therefore, the oxide film on the surface of the Zr-based BMG is main compound of ZrO_2_ and small amounts of Al_2_O_3_, TiO_2_, and Cu_2_O with a thickness around 18 nm.

For CP-Ti, as shown in Figs. S3k and l, the Ti 2*p* spectrum consists of three peaks, at approximately 458.78, 464.46 and 471,15 eV, corresponding to Ti 2*p* for Ti^4+^ oxide state [4], revealing the oxide film on its surface is TiO_2_. Meanwhile, there are no obvious changes of Ti’s chemical state after 150 s, suggesting the thickness of oxide film formed on CP-Ti is approximately 13.5 nm. It is slightly thinner than the Zr-based BMG’s, leading to a plausible interpretation for the better corrosion resistance of the Zr_61_Ti_2_Cu_25_Al_12_ BMG in the electrochemical test (Fig. S3b).

**IV. *In-vitro* cell osteogenic measurements**

Figure S4 displays the images of MC3T3-E1 pre-osteoblasts on the surface of the Zr_61_Ti_2_Cu_25_Al_12_ BMG, CP-Ti and PEEK after culturing for 6 and 24 hours. After 6 hours, the cells showed a typical polygonal osteoblastic shape and well distribution on various specimens. While after 24 hours, more cells can be observed on the Zr-based BMG with extensively spreading and closely contacting.

**Fig. S4.** The morphology of MC3T3-E1 pre-osteoblasts on the surface of the Zr_61_Ti_2_Cu_25_Al_12_ BMG, CP-Ti and PEEK after culturing for 6 hours and 24 hours.

The percentage of living cells (Fig. S5a) and dead cells (Fig. S5b) on the surface of various materials. A significantly higher number of living cells and a lower number of dead cells are found on the surface of the Zr-based BMG compared to CP-Ti (***P* = 0.04 for living and dead cells) and PEEK (****P* = 0.0005 for living and dead cells). Quantification of spreading area on different materials’ surfaces are shown in Fig. S5c. After 24 hours, significant differences take place between the Zr-based BMG vs. CP-Ti and the Zr-based BMG vs. PEEK since their *P* values are 0.006 (**) and 0.01 (*), respectively. The projected cell area (PCA) is shown in Fig. S5d, the larger value represents a more spreading cell morphology. The PCA value of the Zr-based BMG is pronouncedly higher than those of CP-Ti (***P* = 0.002) and PEEK (*****P* < 0.0001). In addition, an integrated optical density (IOD), another statistical factor that indicates a tension of cells, is shown in Fig. S5e. A higher IOD value for the BMG indicates a better cell viability. The Zr-based BMG shows a significantly higher IOD value compared with CP-Ti (***P* = 0.002) and PEEK (*****P* < 0.0001). Meanwhile, CP-Ti shows a larger PCA (***P* = 0.007) and IOD (**P* = 0.012) values compared with those of PEEK. The Optical density (OD) values of various materials at different time points are shown in Fig. S5f. Obviously, the Zr-based BMG presents particularly higher OD values at every time point compared with CP-Ti (*****P* < 0.0001) and PEEK. CP-Ti shows equal OD values as PEEK after 1 and 2 days (*P* > 0.05), which becomes significantly higher after 3 days (*****P* < 0.0001). The morphology information and histogram analysis reveals that the BMG is obviously promoted to cell spreading, which results in better cell adhesion behavior.

**Fig. S5. *In-vitro* cytocompatibility measurements with MC3T3-E1 pre-osteoblast for the Zr_61_Ti_2_Cu_25_Al_12_ BMG, CP-Ti, and PEEK.** The percentage of living cells - **a** and dead cells –**b** on the surface of various materials. **c** The morphology of cells: quantification of spreading area on different materials’ surfaces. Cell adhesion analysis: **d** The projected cell area (PCA). **e** The Integrated optical density (IOD) values of the tested materials. The cell proliferation evaluated by CCK-8 assay: **f** The Optical density (OD) values of various materials at different time points.

**V. *In-vitro* cell angiogenesis measurements**

The percentage of wound healing for the tested materials is shown in Fig. S6a. The Zr_61_Ti_2_Cu_25_Al_12_ BMG has a significantly greater capacity to heal the wound within 24 hours compared with CP-Ti (***P* = 0.01) and PEEK (****P* = 0.0003). The number of HUVECs for each tested material is plotted in Fig. S6b. HUVECs are more numerous in the Zr-based BMG group than in CP-Ti (****P* = 0.0002) and PEEK (*****P* < 0.0001). HUVECs culture with the extract of the Zr-based BMG (Fig. S6c) show significantly more loops formed compared with CP-Ti (*****P* < 0.0001) and PEEK (*****P* < 0.0001). Other parameters, i.e., the covered area (Fig. S6d), total tube length (Fig. S6e), and total branching points (Fig. S6f) in the Zr-based BMG group are also particularly higher than those in CP-Ti (**P* = 0.0324; *****P* < 0.0001; ****P* = 0.0002, respectively) and PEEK (***P* = 0.0039; *****P* < 0.0001; *****P* < 0.0001, respectively). Quantitative histogram analysis demonstrates that the extract of the BMG effectively enhanced an angiogenic ability.

**Fig. S6. *In-vitro* cell (HUVECs, Human umbilical endothelial cells) angiogenesis measurements of the Zr_61_Ti_2_Cu_25_Al_12_ BMG, CP-Ti, and PEEK.** Wound healing assay: **a** The percentage of wound healing for the tested materials. Transwell migration assay: **b** The number of HUVECs for each tested material. Tube formation assay. Quantitative histogram analysis of total loops - **c**, covered area - **d**, total tube length - **e**, and total branching points - **f**.

**VI. Radiographic evaluation**

The femurs were scanned by a high-resolution micro-CT, while the relative details were described in the following: the isolated femurs were putted into a tubular foam sleeve, which can be axially split in half. In order to avoid the influence induced by the increased temperature during radiography, wet wipes were used to cover the sample. Meanwhile, the gap between the sample and the sleeve could be filled up owing to the existence of wipes, which effectively prevented femurs’ moving and turning. Finally, the foam sleeve was folded and fixed with tapes when the consistent orientation of samples was guaranteed.

The area in the radiographic image (Fig. S7a) marked by the yellow dashed line indicates the position of the implant (PEEK is chosen as an example). Figures S7b and c illustrate the region of interest (ROI), which is strictly defined as a column with 1 mm in height and 1.6 mm in diameter. To ensure a consistent and convincing comparison between various materials, we consistently set the ROI at 1.5 mm above the growth plate of the condyles.

**Fig. S7. High-resolution micro-CT evaluation.** A radiographic image of bone and the inserted implant (yellow dots indicate the edges of the implant): **a** scout view. **b** The region of interest (ROI) for micro-CT analysis is defined as a column with 1 mm in height, set at 1.5 mm above the growth plate of the condyles. **c** The ROI is strictly defined as a column of 1.6 mm in diameter.

**VII. Immunofluorescence analysis and Imaging of blood vessels**

The density of Col-I positive structures is shown in Fig. S8a. The Zr-based BMG group shows more positivity for Col-I than CP-Ti (**P* = 0.0049) and PEEK (****P* = 0.0008) groups. The density of CD31 positive structures (Fig. S8b) displays that the Zr-based BMG shows more positivity for CD31 compared with CP-Ti (**P* = 0.02) and PEEK (****P* = 0.0003).

**Fig. S8. Immunofluorescence analysis of the Zr_61_Ti_2_Cu_25_Al_12_ BMG, CP-Ti, and PEEK. a** The density of Col-I positive structures. **b** The density of CD31 positive structures.

**VIII. Element analysis at the implant-bone interface**

In order to characterize the element content at the interface after 6 weeks’ implantation, the analysis on dissected femoral condylar cross sections in the Zr-based BMG group was performed by SEM-EPMA. Figure S9 shows the content of Zr, Ti, Cu, Al and Ca cross the intramedullary the Zr-based BMG and femoral condylar. The present of Ca is significantly higher than other elements at the per-implant region, and its content extremely increased from the interface to the bone. In contrast, the concentration of Zr, Ti, Cu and Al fells off a cliff at the implant-bone interface as indicated by the yellow arrow. Their contents are extremely low and without accumulation in per-implant bone.

**Fig. S9. The element analysis of Zr, Ti, Cu, Al and Ca on dissected femoral condylar cross section in the Zr_61_Ti_2_Cu_25_Al_12_ BMG. a** The SEM image of the cross section. **b** The element contents corresponding to the SEM image.

**IX. *In-vivo* ICP analysis**

Figure S10 displays the *in-vivo* ICP results, i.e., the respective Zr, Ti, Cu and Al ionic concentration in the collected rat blood of the Zr_61_Ti_2_Cu_25_Al_12_ BMG, CP-Ti and PEEK after severing for 2, 4 and 6 weeks. Some differences are detected, e.g. the concentration of Zr and Ti ions at 4 weeks and Al at 6 weeks in the Zr-based BMG group are higher compared with those in CP-Ti and PEEK group, but without significant difference (*P* > 0.05). In spite of this, there is no obviously difference discovered of various ions and materials at all time points (*P* > 0.05). Therefore, systemic changes or abnormal accumulation of Zr, Ti, Cu and Al ions are not induced from the Zr-based BMG in *in-vivo* ICP analysis. The concentration of these ions in blood is far below the corresponding ionic concentration measured in organs studied by Hiroto et al [7].

**Fig. S10. The *in-vivo* ICP results, i.e, the ionic concentration in the collected rat blood of the Zr_61_Ti_2_Cu_25_Al_12_ BMG, CP-Ti and PEEK after severing for 2, 4 and 6 weeks. a** Zr. **b** Ti. **c** Cu. **d** Al.

**X. Hematological analysis**

The systemic hematological analysis, routine blood test and biochemical test are plotted in Fig. S11 and Fig. S12, of rat with the Zr_61_Ti_2_Cu_25_Al_12_ BMG, CP-Ti and PEEK after 2, 4 and 6 weeks’ implantation. CP-Ti and PEEK are served as the references to show the baseline and standard deviation of detected parameters in blood. As shown, pronounced differences are took place between some groups, but they are not biologically meaningful as they are all within the range of random biological variations.

**Fig. S11.** Routine blood test in animals with the implanted Zr_61_Ti_2_Cu_25_Al_12_ BMG, CP-Ti, and PEEK specimens as a function of time. The green area represents the normal range. K/uL and M/uL indicate 10^3^/uL and 10^6^/uL, respectively.

**Fig. S12.** Biochemical parameters in animals with the implanted Zr_61_Ti_2_Cu_25_Al_12_ BMG, CP-Ti, and PEEK specimens as a function of time. The green area represents the normal range.

**References**

[1] G.l. Song, Control of biodegradation of biocompatable magnesium alloys. Corr. Sci.49 (4) (2007) 1696-1701.

[2] C. Stähli, M. James-Bhasin, A. Hoppe, A.R. Boccaccini, S.N. Nazhat, Effect of ion release from Cu-doped 45S5 Bioglass^®^ on 3D endothelial cell morphogenesis. Acta Biomater. 19 (2015) 15-22.

[3] A. Gebert, U. Wolff, A. John, J. Eckert, Corrosion behaviour of Mg_65_Y_10_Cu_25_ metallic glass. Scripta Mater. 43 (3) (2000) 279-283.

[4] http://www.nist.gov/srd/index.cfm.

[5] X.D. Yan, J.L. Sun, Y.N. Meng, Experimental insight into the chemical corrosion mechanism of copper with an oil-in-water emulsion solution. RSC ADV. 8 (18) (2018) 9833-9840.

[6] K. Asami, K. Hashimoto, S. Shimodaira, XPS determination and the compositions of alloy surface and surface oxides on mechanically polished iron–chromium alloys, Corr. Sci. 17 (9) (1977) 713-723.

[7] H. Ida, M. Seiryu, N. Takeshita, M. Iwasaki, Y. Yokoyama, Y. Tsutsumi, E. Ikeda, S. Sasaki, S. Miyashita, S. Sasaki, T. Fukunaga, T. Deguchi, T. Takano-Yamamoto, Biosafety, stability, and osteogenic activity of novel implants made of Zr_70_Ni_16_Cu_6_Al_8_ bulk metallic glass for biomedical application. Acta Biomater. 74 (2018) 505-517.
